# Significance of the intraindividual variability of HLA IgG antibodies in renal disease patients observed with different beadsets monitored with two different secondary antibodies on a Luminex platform

**DOI:** 10.1007/s12026-018-9027-2

**Published:** 2018-10-16

**Authors:** Mepur H. Ravindranath, Edward J. Filippone, Grace Mahowald, Carly Callender, Adarsh Babu, Susan Saidman, Soldano Ferrone

**Affiliations:** 1 Terasaki Research Institute, Los Angeles, CA 90064 USA; 2 Division of Nephrology, Department of Medicine, Sidney Kimmel Medical College at Thomas Jefferson University, Philadelphia, PA USA; 3 Department of Pathology, Massachusetts General Hospital, Harvard Medical School, Boston, MA USA; 4 Immucor Inc., 3130 Gateway Dr, Norcross, GA USA; 5 CSRL, University Hospitals Coventry and Warwickshire, Clifford Bridge Road, Coventry, UK; 6 Department of Surgery, Massachusetts General Hospital, Harvard Medical School, Boston, MA USA

**Keywords:** Calculated panel reactive antibody (cPRA), β2-microglobulin, FcMonoIgG, Heavy chain, Human leukocyte antigen, IgHPolyFab: MFI: mean fluorescent intensity (MFI), Single antigen bead assays (SAB), Denatured antigen

## Abstract

The accurate measurement of anti-HLA alloantibodies in transplant candidates is required for determining the degree of sensitization and for the listing of unacceptable antigens for organ allocation. Both the configuration of the HLA molecules coated on the beads and the nature of detection antibodies may impede assessment of the presence and strength of anti-HLA IgG- with the Luminex single-antigen-bead assay. Sera antibodies of the end-stage renal disease patients were compared using LIFECODES (LC) and LABScreen (LS) beadsets monitored with polyclonal-Fab (*IgHPolyFab*) and monoclonal-IgG (*FcMonoIgG*) second antibodies. Positive results at mean fluorescence intensity (MFI) > 500 (at serum dilution 1/10) were used to calculate panel reactive antibody (cPRA) levels. LS-beadsets are coated with monomeric variants in addition to intact HLA antigens with or without peptides, while LC-beadsets are devoid of monomeric variants and with lesser levels of peptide-free heterodimers. Consequently, IgG antibodies against both classes of HLA were reactive to more antigens with LS than with LC-beadsets. For both classes, MFIs were also frequently higher with LS than with LC. For HLA-I, MFIs were higher with *IgHPolyFab* than with *FcMonoIgG* with the exception of sera with MFIs > 5000 where they were comparable*.* For HLA-II, the reverse occurred, with significantly higher levels with *FcMonoIgG* regardless of the beadsets. The intraindividual variability observed between beadsets with two detection antibodies elucidates that antigens found as acceptable with one beadset may end up unacceptable with the other beadsets, with the possibility of denying potentially compatible transplants to candidates.

## Introduction

Many renal transplant candidates have IgG antibodies against HLA antigens which, depending on the degree of HLA sensitization, can restrict their access to transplantation. The result is increased morbidity and mortality in this population [1, 2]. Upon transplantation, patients with anti-HLA alloantibodies are at increased risk for adverse outcomes. They include hyperacute, accelerated acute, or acute antibody-mediated rejection and delayed graft function in the short term, and chronic active antibody mediated rejection with reduced graft survival in the long term.

Patel and Terasaki [3] developed the complement-dependent cytotoxicity (CDC) assay, allowing a pretransplantation crossmatch to be performed between a recipient’s serum and donor lymphocytes. This assay has essentially abrogated hyperacute rejection. Cell-based assays such as the CDC crossmatch and flow cytometric crossmatch are still used in histocompatibility laboratories to assess the safety of transplantation.

Solid phase assays have now replaced these cell-based assays for the routine detection of anti-HLA antibodies. HLA antigens are attached to a polystyrene bead, either as a mixture of HLA antigens, a phenotypic panel consisting of the HLA antigens present on a single cell line, or a single HLA antigen per bead. The presence of antibodies can be detected by a secondary fluorescent antibody using either flow cytometry or a Luminex platform [4, 5]. The presence of anti-HLA antibodies reacting with a broad array of HLA antigens can be assessed and compared to the frequency of those antigens in the entire donor pool, the so-called calculated panel reactive antibody (cPRA) [2]. It is imperative that these assays be as accurate as possible. False positive reactions may result in denial of a potentially compatible transplant or could falsely elevate the degree of sensitization to inappropriately disadvantage a patient. False negative results could also result in adverse short- and/or long-term consequences.

Unfortunately, the solid phase assays have significant limitations. The native cell surface HLA class I molecule (HLA-I), which exists as trimer of HLA heavy chain (HC), β2-microglobulin (β2M), and peptide, may be disrupted during the manufacturing process, resulting in beads coated with HLA HCs (devoid of β2M and/or peptide), considered as “denatured”. Antibodies that recognize these disassociated HCs, for example, by binding to epitopes exposed by the loss of β2M, may contribute to clinically irrelevant or “false positive” results [6–9]. Work by Grenzi et al. [10] raises similar concerns for HLA class II (HLA-II) antigens, where the specific conformational pairing of α- and β-chains may determine potentially pathogenic epitope expression.

Two vendors’ beadsets are currently available. The LABScreen (LS) beadsets used to monitor antibodies against HLA-I antigens contain not only HLA-trimers but also free HCs lacking β2m and/or peptide [12, 13]. Conformational variants may also occur in the LS beadsets coated with HLA-II antigens [10]. The presence of variants may impede precise identification of antibodies recognizing native intact HLA and may prevent the true assessment of the strength of the antibodies. However, by examining HLA-I antigens on another vendor’s beadsets, LIFECODES (LC) it was observed that the LC-beadsets are primarily devoid of β2M-free HCs [13] with considerably lesser level of peptide-free heterodimers than that of LS. Therefore, we hypothesized that the LC beadsets may provide a more accurate measure of the presence and strength of IgG antibodies specific for native, intact HLA-I. The clinical relevance of such antibodies is emphasized in several reports suggesting that the antibodies targeting intact HLA-I are predictive of graft failure, while those specific for β2M-free HC HLA-I are not [6–9].

A number of variables can potentially confound the measurement of anti-HLA antibodies. The assay can fail to detect high titer antibodies due to interference from complement and/or IgM [14–18]. In addition, low values could also result if the specific epitope recognized by the anti-HLA antibody is shared by multiple antigens within the beadsets. Recently, we have reported that the secondary antibody reagent used to detect the primary anti-HLA antibody bound to the HLA antigen may also impact the measurement of the strength of the anti-HLA antibodies [19]. Currently, both vendors’ beadsets recommend phycoerythrin-conjugated polyclonal F(ab)_2_ binding to the HC constant region (CH1-CH3) of IgG (One Lambda Inc) or to Fc-gamma (CH2, CH3) (Immucor Inc). As the F(ab)_2_ fragments are polyclonal, they can potentially bind to multiple epitopes on the HC of a single anti-HLA IgG antibody, resulting in a potential signal amplification and possible overestimation of the level of the primary anti-HLA antibody. Such amplification is indeed beneficial for immunohistochemical investigations but may affect the assessment of antibody level in Luminex SAB assays [19].

In response to this, we have documented that the use of an Fc-specific monoclonal IgG Ab (*FcMonoIgG*) may provide a better assessment of anti-HLA titer, as it binds to a single Fc-HC specific epitope and hence at a one-to-one ratio with the primary anti-HLA antibody [19]. We have shown that the MFIs obtained with sera or IgG purified from the sera of normal individuals detected with *IgHPolyFab* is higher than that of *FcMonoIgG.* The higher reactivity of *IgHPolyFab* than that of *FcMonoIgG* is attributed to the lower concentration of serum IgG antibodies. However, with post-transplant sera, the MFI obtained with *FcMonoIgG was* consistently higher than that obtained with *IgHPolyFab* presumably due to the higher titer of serum IgG antibodies [19]. These observations suggest that a lower density of anti-HLA IgG bound to the bead surface when the titer of serum antibodies is low, thereby permitting the binding of *IgHPolyFab* to multiple IgH epitopes and resulting in signal amplification. However, when the antibody production is augmented, and the density of IgG bound to the bead may increase due to aggregation of IgG with or without IgM and immune complex [14–18], rendering it less susceptible to binding by multiple molecules of *IgHPolyFab* [19]. Therefore, when the density of the serum antibodies increases, due to aggregation of IgG on the beads, the HC of the primary IgG may not be accessible to *IgHPolyFab.*

In this study, we have used sera from renal transplant candidates and recipients to ascertain the variability of anti-HLA IgG antibody detection using Luminex single antigen beadsets (SAB) from two different vendors with two different secondary antibodies. We hypothesize that the results of this investigation may provide a better strategy to improve the accuracy of these assays and may enable appropriate assessment of cPRA during allocation of deceased donor organs.

## Material and methods

### Patients sera

Sera of ESRD patients, who are candidates for or recipients of kidney, or combined kidney and liver (MGH-018 & MGH-019) or combined kidney and pancreas (MGH-027) transplants were provided by the Histocompatibility (HLA) Laboratory, Massachusetts General Hospital, Boston), after obtaining necessary consent and IRB (2017P001049) approval. Sera were specifically chosen because they were suspected to have non-clinically relevant allo-HLA reactivity, possibly due to antibody to denatured antigens, although some may also have additional clinically relevant reactivity. All sera were tested at a 1/10 dilution.

### Luminex multiplex single antigen beadset assay

#### Beadsets from different vendors

Sera were monitored for HLA-I and -II reactivities using the Luminex SAB assays as described in detail elsewhere [19–23]. The assay uses dual-laser flow cytometry to distinguish sets of polystyrene beads, with each bead containing fluorochromes of differing intensity embedded within the bead. Each bead is coated with a single recombinant HLA antigen. The SAB used in this investigation are (i) LIFECODES (LC) LSA Class I (Class I Cat # 265100R, Lot # 3005613) and Class II beads Cat. # 265200R, Lot # 3005537) (Immucor, Norcross, GA). (ii) LABScreen (LS) Class I (Cat. # LS1A04, Lot # 10) and Class II beads (Cat # LS2A01, Lot # 12) (One Lambda, Canoga Park, CA). The panel of HLA molecules coated on LS and LC beads are similar but differed with respect to a few antigens. Only the beads carrying identical antigens were compared during analysis. The number of identical antigens for each locus was as follows: HLA-A (*n* = 28), HLA-B (*n* = 43), HLA-C (*n* = 13), HLA-DR (*n* = 32), HLA-DQA1/DQB1 (*n* = 17) and HLA-DPA1/DPB1 (n = 13).

#### Monitoring variants of HLA-I antigens on LC and LS Beadsets

Although we have examined the conformational variants of HLA-I on the two vendors beadsets earlier [12, 13], as discussed previously, lot to lot variations may occur. Previously, we have used Lot # 8 and Lot # 9 of LS and Lot # 12235B^1^ of Immucor [13]. Therefore, we examined the conformational variants on the current lots using the detailed protocol and three unique monoclonal antibodies (mAbs) used in the previous report [12]. The mAbs used include W6/32, HC-10, and TFL-006, all of which belong to the IgG2a subclass. The binding of these mAbs to the HLA on the beads was ere assessed with an IgG2a-specific mAb [12, 13].

#### Protocol differences between vendors and the protocol used in this study

The two vendors provide different protocols for using their respective beadsets, which were previously compared and found to result in minimal difference in MFI between the protocols [13]. To remove protocol differences as a confounding variable, and since a slightly modified version of the One Lambda protocol is the standard procedure in our laboratory [19–23], we have used the modified One Lambda protocol (using 2 μl of beads instead of 5 μl as recommended by the manufacturer) for both LS and LC beadsets [13]. Further details of the protocol differences are provided in our previous report [13]. The bead concentration was similar in both beadsets for 2 μl of beads.

Twenty microliters of diluted (1/10) serum was incubated with 2 μL of beads for 30 min at room temperature (RT), on a shaker. The beads were then washed (3X) with LS Wash Buffer. The antibody binding to beads was assessed with two PE-conjugated secondary antibodies (see below), by incubating the secondary antibody (50 μL at 5 μg/mL) for 30 min at RT on a shaker. After washing, the beads were suspended in 1X PBS before acquisition on the Luminex®. Approximately 100 beads were counted for each antigen.

The SAB assay includes a positive control (coated with human IgG) and negative control (no antigen) beads. In addition, we have used negative control serum (devoid of anti-HLA IgG) as well as positive control serum, prepared by pooling sera from several individuals carrying anti-HLA IgG. The IgG reactivity of each bead was recorded as normalized MFI after normalizing the Trimmed mean MFI values obtained with PBS only, the negative control bead (NC) and then with the mean of the negative sera (NGS) control samples provided with LS and LC kits as follow: *Normalized MFI* = *[(Trim. Mean MFI - PBS MFI) - (NC MFI)] - (NGS-LS + NGS- LC)/2).*

### Diversity in secondary antibody

Two PE-conjugated secondary-antibodies were used in this study. PE-conjugated affinity purified human IgG HC (IgH) binding polyclonal goat-anti-human IgG antibody fragments [F(ab)_2_] (*IgHPolyFab*) and human IgG Fc-specific mouse monoclonal IgG (*FcMonoIgG*). *IgGPolyFab* is supplied as 0.5 mg/ml in PBS pH 7.6, by One Lambda Inc. (Canoga Park, CA) Cat # LS-A82. The label on the box of vials provided by *One Lambda Inc (Canoga Park, CA)* clarifies the product as “*PE-conjugated goat-anti-human IgG, R-phycoerythrin-conjugated affini-pure F(ab’)*_*2*_ *goat X-human IgG 1 ml (100X)”. FcMonoIgG* is supplied as 0.5 mg of purified IgG in 1 ml of borate buffered saline (pH 8.2) by *Southern Biotech* (Birmingham, AL) and “reacts with the Fc portion of the HC of all subclasses of Human IgG.” Further details regarding the secondary antibodies are provided elsewhere [19].

All tests were performed by one individual, all at the same time and in a single tray. The number of HLA-I and -II recognized by each serum and each combination of beadsets and secondary antibodies were determined. In addition, the approximate strength of any detected antibody as measured by normalized MFI was compared, using a MFI cutoff for positivity of 500, at serum dilution 1/10.

### cPRA calculations

For this experimental investigation, the cPRA of the sera were calculated for each vendor beadset and each secondary antibody, using the cPRA calculator on the UNOS website (https://optn.transplant.hrsa.gov/resources/allocation-calculators/cpra-calculator). Any specificities with MFI values greater than or equal to 500 were considered unacceptable antigens. The base antigen group, as well as the specific antigen, was called positive when applicable in the UNOS calculator (e.g., if, the A*02:03 bead is positive both A2 and A0203 were checked off in the calculator). DR51, DR52, and DR53 were checked off if any of the corresponding DRB3, DRB4, or DRB5 beads reacted at or above 500 MFI. The UNOS cPRA calculator is currently unable to consider DQA1, DPB1, or DPA1 antibodies.

### Statistical analysis

Differences in the number of positive antigens between LC and LS beadsets and secondary antibodies *FcMonoIgG IgHPolyFab*) did not follow a normal distribution, therefore, non-parametric *p* values were computed to assess their level of difference. Similarly, the MFI values of different IgG antibodies reacting to different HLA antigens showed differences between the above test parameters. Paired comparisons of the number of antigens recognized, as well as the MFI, are made (i) between LC versus LS for both secondary antibodies (ii) between *FcMonoIgG* vs *IgHPolyFab* for both beadsets.

## Results

### Assessment of conformational variants

**Fig. 1:**
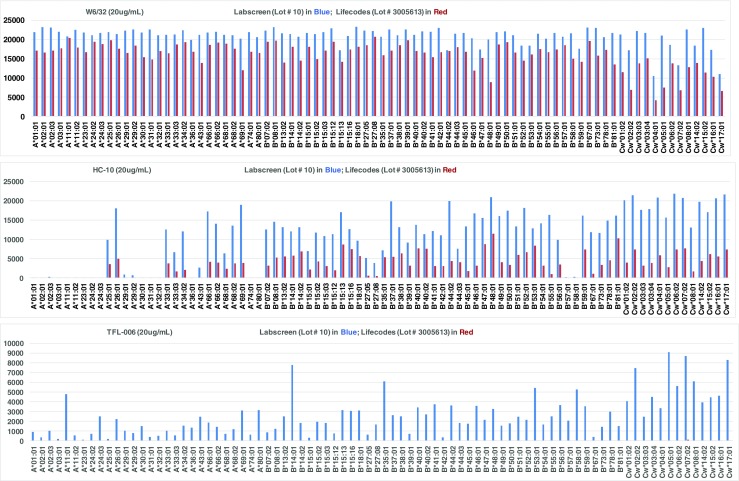
Reactivity of Anti-HLA-I mAbs (W6/32, TFL-006, HC-10) against all HLA-A (*n* = 28), HLA-B (*n* = 44) and HLA-C (*n* = 13) antigens coated on LS (Cat # LS1A04, Lot 10) (Blue) and LC (Cat # 265100R, Lot# 3005613) (Red) HLA-I beadsets. The MFI cutoff used for a positive reaction is 1000. Note that W6/32 bound to 100% of antigens on both beadsets. HC-10 bound to fewer HLA-A antigens (10/28 for LC and 13/28 for LS), most HLA-B antigens (95%) and all HLA-C antigens (100%). In contrast, TFL-006 bound to most of the alleles on LS but failed to bind to any antigens on LC beadsets, confirming the paucity of β2 m-free HLA heavy chains in LC

The results include assessment of (1) the number of HLA class I and II antigens recognized and (2) the strength or level of antibodies reacting to these antigens measured as MFI. Our examination of the beadsets with the three monoclonal antibodies confirmed that the new LS beadsets had all the conformational variants [12, 13]. The new LC beadsets had β2m-associated HLA mostly with peptides but to a lesser extent with HLA devoid of peptides, as assessed by HC-10. With mAb TFL-006, which identifies β2 m-free HLA HC, LC, but not LS beadsets, remained negative (Fig. 1). This investigation is restricted to the HLA antigens common to both beadsets and does not include values obtained with the antigens specific for each beadset, which are summarized in Table 1. Conformational variants on HLA-II beads were not tested, for no protocol has been designed as was done for HLA-I beads [14, 15].

**Table 1:** Reactivity of ESRD patients’ sera to the unique HLA antigens coated on LC and LS beadsets. Each beadset have several unique HLA-I and HLA-II antigens not found in other vendor’s beadsets. Antigens on the beadsets were detected with two different secondary antibodies, namely *FcMonoIgG* &* IgHPolyFab.* Sera IDs are given if an antigen is reactive; Antigens recognized by one of the secondary antibodies is indicated as Mono + or Poly +

HLA-Class I Beadsets			
Unique to LIFECODE (*n* = 12)	MGH sera	Unique to LABScreen (n = 12)	MGH sera
A*02:02	008/015 (015 only Poly +)	A*02:06	008/15
A*02:05	[008]	A*30:02	(007 only Poly+]
B*07:03	None	A*34:01	[007]
B*15:18	None	B*13:01	None
B*27:03	None	B*15:10	None
B*35:08	None	B*40:06	None
B*82:01	[007]	B*51:02	001/7/27, all Poly+
Cw*04:03	002/007/008 (MGH-002 only Mono +)	B*57:03	001/2/8/11/27 (001 only Poly+)
Cw*07:01	None	B*82:02	None
Cw*08:02	None	Cw*03:02	[007]
Cw*12:02	[007]	Cw*12:03	007/024, (024 only Poly+)
Cw*18:01	None	Cw*18:02	007/25
HLA-Class II Beadsets			
Unique to LIFECODE (*n* = 34)	MGH sera	Unique to LABScreen (*n* = 34)	MGH sera
DRB1*03:03	(007 o + S15:S41nly Poly +)]	DRB1*09:02	001/018/019/027] (011/019/027 only Mono*)
DRB1*08:02	007/011	DRB1*14:02	[007]
DRB1*11:03	007/011	DRB1*14:54	[007]
DRB1*13:05	007/011	DRB4*01:03	005/018/023
DRB1*14:03	007/011 (007 only Poly+)	DQB1*02:01\DQA1*03:01	00100/6/008/018/027 (001/006 only Mono+
DRB1*14:04	[007]	DQB1*02:01\DQA1*04:01	007/018
DQB1*02:02\DQA1*03:02	008/018	DQB1*03:01\DQA1*02:01	007/008/011/018
DQB1*02:02\DQA1*05:01	007/018	DQB1*03:03\DQA1*03:01	None
DQB1*03:01\DQA1*03:02	007/008/011	DQB1*03:01\DQA1*05:03	007/008/011
DQB1*03:01\DQA1*05:01	007/008/011	DQB1*03:01\DQA1*05:05	007/008/011
DQB1*03:03\DQA1*04:01	007/008/011	DQB1*03:03\DQA1*02:01	007/008/011/018
DQB1*03:03\DQA1*06:01	007/008/011	DQB1*04:01\DQA1*03:03	007/008
DQB1*04:01\DQA1*04:01	[007]	DQB1*04:02\DQA1*02:01	None
DQB1*04:01\DQA1*05:01	[007]	DQB1*06:02\DQA1*01:01	008/124 (024 only Poly+)
DQB1*04:02\DQA1*03:01	007/009	DQB1*06:09\DQA1*01:02	[008]
DQB1*04:02\DQA1*06:01	[007]	DPB1*03:01\DPA1*01:05	None
DQB1*05:01\DQA1*01:02	[008]	DPB1*03:01\DPA1*02:01	[024 only Poly+]
DQB1*05:03\DQA1*01:04	[008]	DPB1*04:01\DPA1*01:03	None
DQB1*06:01\DQA1*01:04	[008]	DPB1*06:01\DPA1*02:01	None
DQB1*06:01\DQA1*02:01	008/018	DPB1*10:01\DPA1*02:02	None
DPB1*01:01\DPA1*02:02	None	DPB1*11:01\DPA1*01:03	[024 only Poly+]
DPB1*01:01\DPA1*03:01	None	DPB1*11:01\DPA1*02:02	[010/015/024 (024 only Poly+)]
DPB1*04:01\DPA1*01:03	None	DPB1*13:01\DPA1*02:02	[001/005/006/016 all Mono+, [024]
DPB1*04:01\DPA1*02:01	None	DPB1*13:01\DPA1*03:01	[024 only Poly+]
DPB1*04:01\DPA1*02:02	None	DPB1*18:01\DPA1*01:04	None
DPB1*04:01\DPA1*03:01	None	DPB1*18:01\DPA1*01:05	None
DPB1*04:01\DPA1*04:01	None	DPB1*18:01\DPA1*02:01	None
DPB1*04:02\DPA1*03:01	None	DPB1*19:01\DPA1*01:03	[002/006/016/024/027] (002/006/027 Mono+)
DPB1*05:01\DPA1*03:01	None	DPB1*09:01\DPA1*02:01	None
DPB1*11:01\DPA1*02:01	None	DPB1*20:01\DPA1*03:01	None
DPB1*13:01\DPA1*04:01	None	DPB1*23:01\DPA1*02:01	001/002/019/020/023/025, (002/025 Mono+)
DPB1*18:01\DPA1*01:03	None	DPB1*28:01\DPA1*01:03	None
DPB1*19:01\DPA1*02:01	None	DPB1*28:01\DPA1*01:05	None
DPB1*28:01\DPA1*02:02	None	DPB1*28:01\DPA1*04:01	None

### Intraindividual disparity in the number of HLA antigens recognized by patients’ sera

#### Difference in the number of HLA-I antigens recognized by patients’ sera

The number of HLA-I antigens (HLA-A/-B/-C) recognized by different sera (*n* = 15) when tested with both secondary antibodies with the two beadsets is presented in Table 2. No antibody level (MFI < 500) was detectable in the sera MGH-005/-023/025 with both beadsets and secondary antibodies.

**Table 2:**
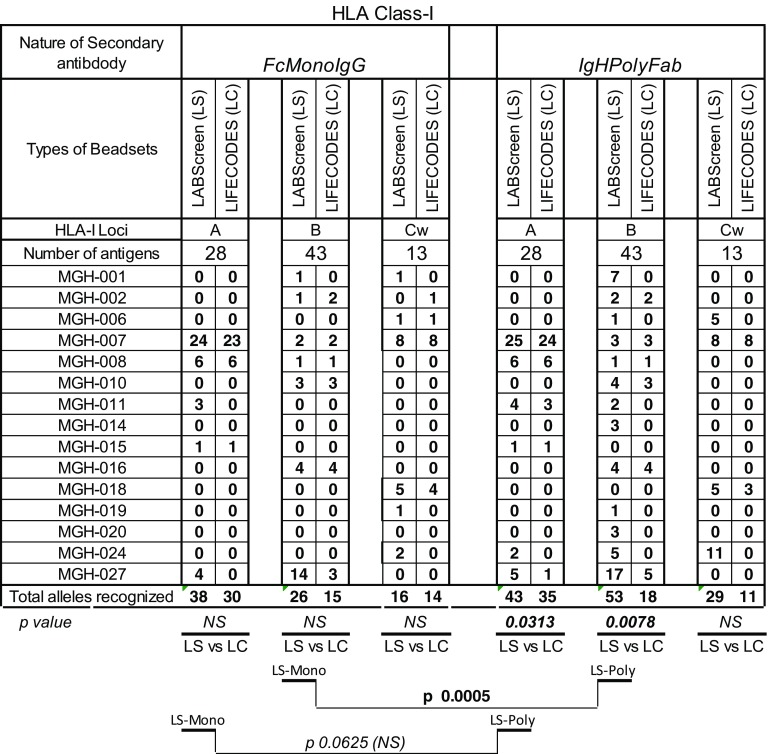
Intraindividual disparity in the number of HLA-I antigens recognized by patient sera between different secondary antibodies and beadsets

Significant *p* values are provided. 0 values refer to absence of reactivity. Three sera that had no reactivity with any beads (data are not shown). Beadsets specific antigens are not included in the study. The sera were tested at 1/10 dilution and the cutoff of the normalized MFI is > 500

The number of antigens detected with all other sera is higher with LS than with LC as follows: for HLA-A (with *IgHPolyFab* 4 sera LS > LC, 2 LS = LC, 0 LS < LC; with *FcMonoIgG* 3 LS > LC, 2 LS = LC, 0 LS < LC)) for HLA-B (with I*gHPolyFab* 9 LS > LC, 4 LS = LC, 0 LS < LC; with *FcMonoIgG* 2 LS > LC, 4 LS = LC, 1 LS < LC) for HLA-Cw (with I*gHPolyFab* 3 LS > LC, 1 LS = LC, 0 LS < LC; with *FcMonoIgG* 4 LS > LC, 2 LS = LC, 1 LS < LC). Taking all sera into consideration, with *IgHPolyFab,* the total number of antigens recognized by the antibodies for HLA-A (*p* < 0.0313) and HLA-B (*p* < 0.008) were significantly higher for LS than for LC. There is no significant difference in the total number of antigens recognized by antibodies for any loci, with LC tested with different secondary antibodies.

#### Difference in the number of HLA-II antigens recognized by the sera

The number of HLA-II antigens (HLA-DR/DQ/DP) recognized by different sera (*n* = 15) when tested by the two beadsets and secondary antibodies beadsets is presented in Table 3. Antibodies were not detectable (MFI < 500) in the sera MGH-0014/-016/-20 with both beadsets and secondary antibodies. The number of antigens detected with all other sera is higher with LS than with LC as follows: for HLA-DR (with *IgHPolyFab* 6 sera LS > LC, 2 LS = LC, 6 LS < LC; with *FcMonoIgG* 8 LS > LC, 5 LS = LC, 2 LS < LC) for HLA-DQ (with *IgHPolyFab* 1 LS > LC, 3 LS = LC, 1 LS < LC; with *FcMonoIgG* 4 LS > LC, 4 LS = LC, 1 LS < LC) for HLA-DP (with *IgHPolyFab* 3 LS > LC, 0 LS = LC, 0 LS < LC; with *FcMonoIgG* 2 LS > LC, 0 LS = LC, 0 LS < LC;). Taking all sera into consideration, with *FcMonoIgG,* the total number of antigens recognized by the antibodies for HLA-DR (*p* < 0.01) and HLA-DP (*p* < 0.0313) were significantly higher for LS than for LC; with *IgHPolyFab,* the total number of antigens recognized by the antibodies were also higher for LS for all three loci.

**Table 3:**
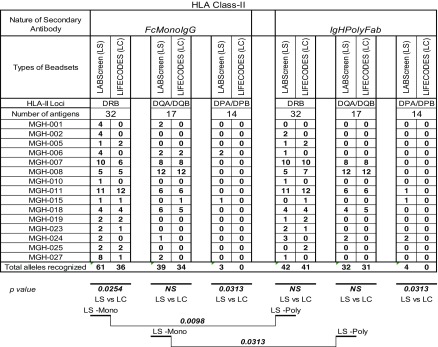
Intraindividual disparity in the number of HLA-II antigens recognized by patients’ sera between different secondary antibodies and beadsets

Significant *p* values are provided. 0 values refer to absence of any reactivity. Beadsets specific antigens are not included in the study. The sera were tested at 1/10 dilution and the cutoff of the normalized MFI is > 500

### Intraindividual disparity in the MFI levels of anti-HLA IgG

#### Significant differences in the MFI levels of anti-HLA-I IgG between the two beadsets and the two secondary antibodies.

Based on the number of HLA-I antigens (> 5 versus < 5) recognized by the antibodies, the sera are categorized into two groups: Group 1 consists of sera (*n* = 8) reacting to > 5 HLA antigens, ranging in the number of antigens from 5 to 35. Group 2 consists of sera (*n* = 7) reacting to <5 HLA antigens. Due to small sample size (< 5) of antigen recognition, Group 2 did not show any statistically significant differences in MFI between beadsets or secondary antibodies. The Group 1 antibody profiles revealed three major patterns of HLA reactivities (Table 4). Group 1A **(**MGH-001 & MGH-024**):** Mostly MFI (< 1000) observed with one of the two beadsets with one or both secondary antibodies. Group 1B (MGH-006 & MGH-011): More antigens showed MFI (> 1000) in any one of the beadsets, while the other showed either low MFI (< 1000) or not more than one antigen with high MFI (> 1000). Group 1C (MGH-007, MGH-008, MGH-018 & MGH-027): Many antigens showed highest MFI (> 1000) consistently with both beadsets and with both secondary antibodies.

**Table 4:**
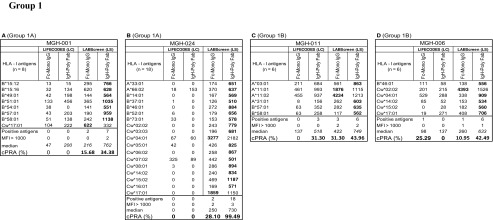

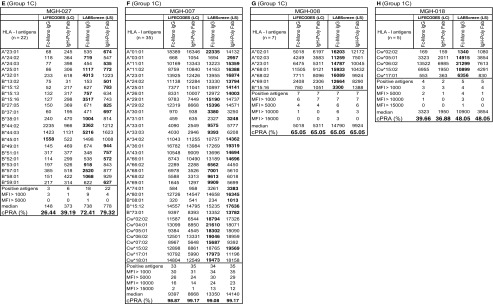

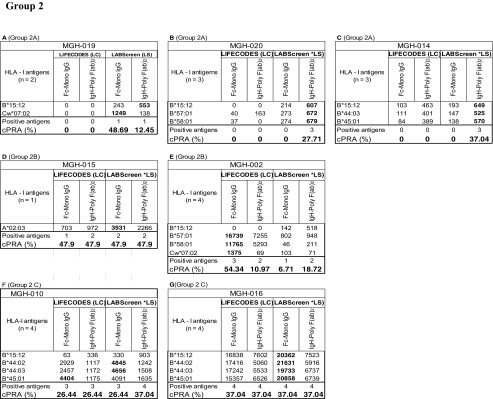
Intraindividual disparity in the MFI of HLA-I reactive antibodies (Group 1 against > 5 HLA antigens, Group 2 against < 5 antigens) in patients’ sera using different secondary antibodies and different beadsets

Based on the MFI values (MFI > 500 = positive), percentage cPRA were determined using the UNOS cPRA calculator (https://optn.transplant.hrsa.gov/resources/allocation-calculators/cpra-calculator). Groups are as described in the “Results” section. Bold MFI values under both beadsets refer to the higher MFI observed among the four categories

Group 1A (MGH-001 & MGH-024): Detection of HLA antibodies with positive MFI values (in bold in Table 4) only with the LS beadsets is a striking feature of this group. The positive values are consistently higher with *IgH PolyFab* than with *FcMonoIgG*. However, in both sera, antibodies reacting to Cw*17:01 is higher in LS with *FcMonoIgG* than with *IgHPolyFab* and the most predominant antibody in MGH-024 sera reacting with the LS beadset is Cw*04:01 detected with *FcMonoIgG*. Both sera did not show any reactivity (MFI < 500) with LC beadsets with both secondary antibodies.

Group 1B (MGH-006 and MGH-011): While most of the antibodies with positive MFI were observed with LS, as in Group 1A, one or two antibodies were also observed with LC. Sera showed higher MFI with LS than with LC (in bold in Table 4). With LS beads, although reactivity to several antigens was higher with *IgHPolyFab*, reactivity to one (MGH-006; Cw*02:02) or two (MGH-011; A*11:01, A*11:02) antigens showed the very high MFI with *FcMonoIgG. IgHPolyFab* showed reactivity to the antibodies bound to these antigens along with those reacting to A*03:01 on LC beadsets. Antibodies to A*03:01, A*11:01 and A*11:02 on LC were recognized only by *IgHPolyFab*. Interestingly, MGH-011 showed low reactivity to A*31:01, B*57:01 and B*58:01 only with *IgHPolyFab* on LS beadsets. Similar reactivity was noted with MGH-006 on LS detected only with IgHPolyFab for Cw*14:02, Cw*16:02 and Cw*17:01 and B*46:01.

Group 1C (MGH-007, MGH-008, MGH-018 & MGH-027): Three (MGH-007/-008/-018) of the four sera showed very HLA high reactivity (MFI > 10,000) with both LS and LC and with both secondary antibodies. MGH-007 showed reactivity to 35 HLA-I antigens on both LS and LC beadsets with *IgHPolyFab*. Although similar reactivity is observed with *FcMonoIgG,* reactivity to one or two antigens remained negative on both beadsets (e.g., B*08:01). MGH-027 showed positive MFI against 22 antigens. Antibodies detected by both secondary antibodies on both beadsets were reactive to B*44:02, B*44:03, B* 45:01. Higher MFI levels were observed with *FcMonoIgG* with both beadsets (see MFI in bold in Table 4). All antigens recognized by MGH-008 (*n* = 7) and MGH-018 (*n* = 5) were positive with both beadsets and both secondary antibodies with minor variations. In contrast with the other sera, higher MFI levels were observed when using *FcMonoIgG* as the secondary antibody. For group 1 overall, in groups 1A and 1B higher MFI was observed with *IgHPolyFab* than with *FcMonoIgG;* in two of the sera in group1C (MGH-7 and MGH-27), the MFI levels are almost equal with both the secondaries, but in the other two (MGH-8 and MGH-18), the MFI levels are higher with *FcMonoIgG*.

Analysis of the anti-HLA-I antibody profiles of Group 2 as detected by the combinations of different beadsets and secondary antibodies also revealed three major patterns of HLA reactivities (Table 4).

Group 2A **(**MGH-014, MGH-019 & MGH-020**):** Mostly MFI is positive only with LS and that too with only with *IgHPolyFab*. *FcMonoIgG*, which reacted only with MGH-019, showed high MFI (1249) for Cw*07:02 on LS only. Interestingly, the mAb TFL-006 which recognizes β2 M-free HC showed maximum binding (> 70% of mAb W6/32 binding) with Cw*07:02 on the LS (Fig. 1). All sera were reactive to HLA antigen B*15:12 on the LS but not on LC and that too only with *IgHPolyFab*.

Group 2B (MGH-002 & MGH-015): Antigens show high MFI (> 1000) in any one of the beadsets, while the other showed either low MFI (< 1000) or not more than one antigen with high MFI (> 1000). MGH-002 showed MFI higher than > 10,000 for B*57:01 and B*58:01 with *FcMonoIgG.* MFI higher than > 1000 is noted for Cw*07:02 with LC beadsets, when tested with *FcMonoIgG. It may be recalled that LC beadsets are totally negative for TFL-006,* which recognizes β2 M-free HC. Therefore, in contrast to Cw*07:02 reactivity of MGH-019 (Group 2B), the reactivity of MGH-002 on LC may signify the presence of an intact native form of Cw*07:02.

Group 2C (MGH-010 & MGH-016): Many antigens showed highest MFI (>1000) consistently with both beadsets and with both secondary antibodies. Both sera showed reactivity to B*15:12. The highest MFI is observed for B*45:01 with LC beadsets tested with *FcMonoIgG.*

In summary, with sera groups 1A, 1B & 1C, higher MFI values are observed with LS compared with LC. Additionally, the MFI values also differed between the two secondary antibodies. Statistical analysis of group 1A-C is presented in Table 6 confirms the following:

1. The median MFI of HLA-I antibodies are higher in all three groups with LS than with LC.
2. The median MFI of the antibodies are higher for Groups 1A and 1B on both LS and LC when tested with *IgHPolyFab*. In both these groups, MFI of antibodies for any HLA antigen when tested with *IgHPolyFab* rarely exceeded 2000. For Group 1C, the MFI values were often higher than 2000 (exception MGH-027), and the individual MFI of HLA antigens on both LS and LC were higher when tested with *FcMonoIgG.*

Thus, the results obtained with antibodies to HLA-I antigens, provide evidence that the fluoresceinated secondary antibody is an important factor contributing to intraindividual variability in the MFI. On LS beadsets*, IgHPolyFab* showed higher median MFI than *FcMonoIgG for* six of the eight sera enumerated above. The median MFIs of five sera (MGH-001/-024 (Group 1A); MGH-006/-011 (Group 1B); MGH-027 (Group 1C) recognized by *IgHPolyFab* were > 500 but < 2000 when tested at 1/10 dilution. However, when tested with *FcMonoIgG* or on LC beadsets when tested with both secondary antibodies, the MFIs were mostly negative (MFI < 500) for these five sera. Therefore, what had been recognized as an “unacceptable antigen” by *IgHPolyFab* on LS beadsets, may represent false positive reactivity. The above suggestion requires further validation by flow cross matches with antigen positive cells. In contrast, sera with high reactivity such as MGH-008, MGH-018, which had a median MFI > 5000 were recognized by *FcMonoIgG better* than *IgHPolyFab.* Since both *FcMonoIgG and IgHPolyFab* show high MFI, there will be no difference in acceptability.

#### Intraindividual disparity in the MFI levels of anti-HLA-II IgG recognized by patients’ sera

The HLA-II antibody profiles also fall into two categories. Group 1 consists of sera (*n* = 8) reacting to > 5 HLA antigens, ranging from 5 to 35. Group 2 (*n* = 7 sera) consists of sera reacting to < 5 HLA antigens. No statistical inference between beadsets or secondary antibodies could be made due to a low number (*n* = 5) of positive antigens in Group 2. Examination of the anti-HLA-II antibody profiles of Group 1, as detected by the combinations of different beadsets and secondary antibodies reveal three major patterns of HLA reactivities (Table 5). The groups are based on antigens common to both beadsets of both vendors, and the unique beadsets of the two vendors are not included in categorizing the beadsets.

**Table 5:**
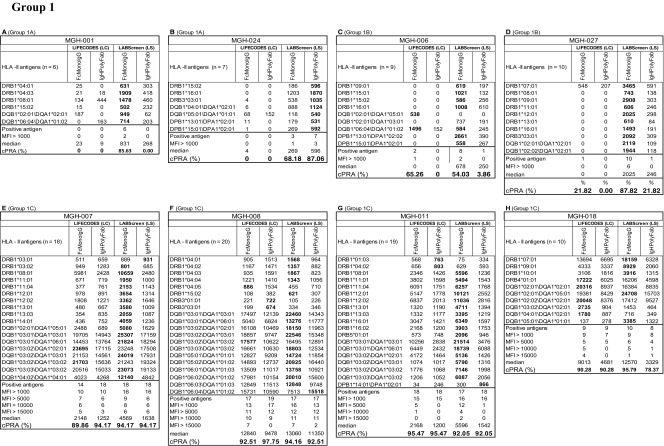

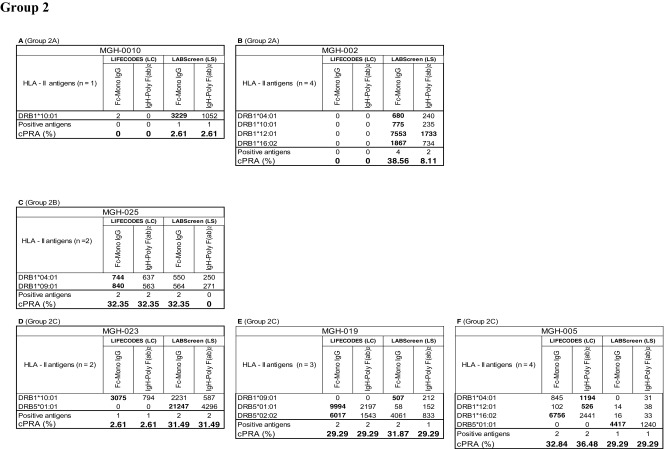
Intraindividual disparity in the MFI of HLA-II reactive antibodies (group 1 against > 5 HLA antigens, group 2 against < 5 antigens) in patients’ sera using different secondary antibodies and different beadsets

Percentage cPRA were determined using the cPRA calculator on the UNOS website (https://optn.transplant.hrsa.gov/resources/allocation-calculators/cpra-calculator). Groups are as described in the “Results” section. Bold MFI values under both beadsets refer to the higher MFI observed among the four categories

Group 1A (MGH-001, MGH-024): In this group, positive MFI values are observed only with LS, while LC beadsets were totally negative. MGH-001 differed from MGH-024, in that higher MFI values (in bold in Table 5) were observed with *FcMonoIgG* but not with *IgHPolyFab*. While the reverse was true for MGH-024 in that the positive MFI values were observed only with *IgHPolyFab*.

Group 1B (MGH-006, MGH-027): Most of the antibodies with positive MFI were observed only with LS, as in Group 1A, with one or two antibodies recognized on LC. In the few cases where antibodies were detected on LC beadsets (two for MGH-006 and one for MGH-007), the MFIs were > 500 only when *FcMonoIgG* was used as the secondary antibody.

Group 1C (MGH-007 (number of antibodies (*n*) = 18), MGH-008 (*n* = 20), MGH-011 (*n* = 19), MGH-018 (*n* = 10). Many antigens showed high MFI (> 1000) consistently with both beadsets and with both secondary antibodies. Frequently, all sera showed the highest MFI values (in bold) with *FcMonoIgG* on LS beadsets.

Analysis of the anti-HLA-II antibody profiles of Group 2 also revealed three major patterns of HLA reactivities (Table 5).

Group 2A (MGH-010 & MGH-002): In both sera, antibodies to DRB antigens with MFI >500 are detected only on LS beadsets tested with *FcMonoIgG,* as secondary antibody.

Group 2B (MGH-025): Antibodies to DRB antigens with MFI > 500 are detected with both LS and LC.

Group 2C (MGH-005, MGH-019 & MGH-023): Many antigens showed high MFI (> 1000). There are some antibodies that are detected by both LS and LC beadsets (MGH-023 and MGH-019), while some are only detected on LS (MGH-23, MGH-019, and MGH-005) and some on only LC (MGH-005). Highest MFI is observed for one or two alleles in all sera with LC beadsets tested with *FcMonoIgG.* All sera recognized DRB antigens only.

In summary, with groups 1A-C, higher MFI values are observed with LS compared to LC. Similarly, the MFI values differed between the two different secondary antibodies. Statistical analysis presented in Table 6 confirms the following:

1. The median MFI of sera in all the three groups (1A- C) (with the exception of MGH-018) are significantly higher for the anti-HLA-II IgG antibodies with LS than with LC beadsets.
2. The median MFI obtained with LS beadsets are higher for *FcMonoIgG* than for *IgHPolyFab*. This is particularly true for group 1C (the high MFI group). The median MFI of the anti-HLA-II antibodies observed for both beadsets with the two different secondary antibodies were consistently lower than 2000 for groups 1A & 1B (Table 6) in contrast to Group 1C for both beadsets and for both secondary antibodies.

**Table 6:**
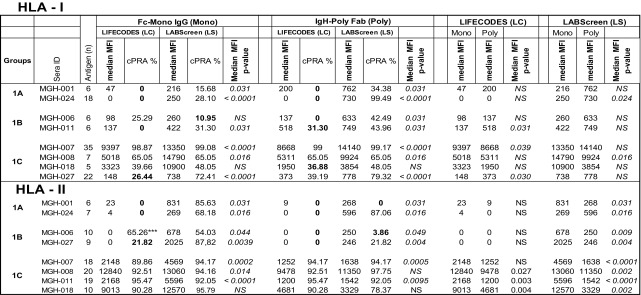
Percentage cPRA values in relation to median MFI of HLA-I and HLA-II antigen reactive antibodies in patients’ sera using two beadsets and two secondary antibodies. The low value among different combinations of beadsets and secondary antibody shows that the use of the beadset or secondary antibody may avoid denial of deceased donor organ for the recipients. Sera groups 2A-C of HLA-I and HLA-II antigens are not included since the positive antigen sample size is < 5 and the *p* values were insignificant. Low cPRA values are shown in bold.

***Two DQ antige*NS* showed high MFI only in LC (see Table 5).  Italicized  symbols refer to statistical significance

Thus, the median MFIs for HLA-II antigens are generally higher with *FcMonoIgG* than with *IgHPolyFab* (13 out of 14 sera tested). A subset of sera (MGH-001/006/024/027) displayed a pattern in which reactivity was present predominantly with LS but absent with LC beadsets. A notable exception to this is the reactivity seen in MGH-006 against the DQB1*06:01/DQA1*01:02 and DQB1*02:01/DQA1*05:01 beads from the LIFECODES beadset. The sera of group 1C (MGH-007/−008/−011/−018), which had median MFIs >2000 with *FcMonoIgG* on both beadsets, generally showed higher MFI on LS.

### Intraindividual disparity in the MFI of anti-HLA serum IgG reflects the disparity in cPRA

Of the three groups of sera (Group 1A-C) reacting to both HLA-I and HLA-II antigens, groups 1A and 1B document a high level of intraindividual disparity based on the beadsets and secondary antibodies, which is reflected in the cPRAs. High median MFIs of antibodies reacting to HLA-I antigens, as well as the corresponding high percentage cPRA, are shown in Table 6**.** The high percentage of cPRA correlates with LS beads when tested with *IgHPolyFab* for HLA-I. For class II, the high percentage cPRA correlated well with the high median MFIs on LS beads tested with *FcMonoIgG.* However, there were few exceptions. For example, the MFI observed with MGH-024 sera (Group 1A) was higher with *IgHPolyFab* and so also was the cPRA. However with MGH-006 sera (a Group 1B), although the median MFI was 0, LC showed the highest cPRA due to a single allele (shown with asterisks).

In summary, as shown in Table 6, the higher median MFIs observed with LS beadsets paralleled with high percentage cPRAs, when tested with *IgHPolyFab* for HLA-I and with *FcMonoIgG* for HLA-II. Higher percentage (e.g., > 30%) of cPRA obtained using LS beadsets for HLA-I, when tested with *IgHPolyFab* and HLA-II, when tested with *FcMonoIgG* may suggest the greater number of unacceptable antigens.

## Discussion

The current Luminex single antigen bead (SAB) assay used to screen for anti-HLA antibodies is not a quantitative assay [11, 24]. Despite the fact, the assay has received enormous clinical attention, since it can efficiently detect antibodies in a specific and sensitive manner that is not achievable by other methods. In this investigation, we address some of the technical issues in the measurement of the MFIs that arose while assessing intraindividual variabilities in the SAB assay. Obviously, these issues should be clarified prior to extending the assay for quantitation or the clinical evaluation of the antibodies. The intraindividual variabilities emerged while comparing the SAB assays with HLA-coated beadsets from two different vendors using two different secondary antibodies, namely IgH-binding polyclonal Fab and Fc-specific monoclonal IgG. One of the fundamental questions in measuring antibodies against HLA class I or class II antigens is whether the assay detects the antibodies directed against intact or native trimeric (homo- or heterodimers with peptide) HLA or against native HLA admixed with antibodies binding to the monomeric (“denatured”) variants of HLA, which are commonly referred to as “denatured HLA”. This is critical because numerous studies document that antibodies against intact HLA but not those formed against “denatured HLA” are pathogenic [6–9, 26]. There is an imminent need to provide the clinicians and HLA laboratories with beadsets coated only with intact trimeric HLA, devoid of monomeric or “denatured” variants of HLA, using appropriate single primary antibody-binding secondary detection antibody [19].

Recombinant HLA are coated on the beads with the premise that they mimic the native HLA expressed on the cell surface [4, 5]. A native HLA molecule, whether it is HLA-I or HLA-II, consists of two polypeptide chains and peptide, i.e., HC and β2M with exogenous peptide for HLA-I, and α-chain and β-chain with peptide for HLA-II. During preparation and purification of the recombinant HLA molecules, the native configuration may not remain intact as a trimer but can be disrupted while purifying or when coating on the bead surface. The serum antibodies may react with the disrupted or “denatured” HLA molecules, by binding to the “shared” or “common” or “public” epitopes among HLA molecules in addition to some antibodies binding to the unique or specific epitopes characteristic of the native, intact, trimeric HLA. MFI emanating from this combination of antibodies binding to both native and denatured HLA will not truly reflect the density of the antibodies binding to the intact HLA antigens on the allograft. Early investigators distinguished antibodies recognizing native versus “denatured” HLA [11, 24, 25] by subjecting the LS beads to acids, which disrupted the intact HLA or to heat, which altered or coagulated the structure of homo or heterodimers. Recognizing the interference of antibodies reacting to denatured (disrupted) HLA, the vendor of LS beadsets, namely One Lambda Inc., modified LS beadsets to generate an unique beadsets without conformational or denatured variants, called iBeads [8, 9, 22, 26]. Visentin et al. [9, 26] compared the antibody profiles recognizing iBeads versus the standard LS beadsets and inferred that sera may contain (a) anti-HLA antibodies reacting to native intact HLA only, (b) “denatured” or dissociated HLA only, and (c) those reacting to the epitopes of both native and denatured HLA. However, the commercial production of iBeads were abandoned, in spite of its potential usefulness in the light of the findings that the antibody against the native intact HLA-I were pathogenic, while those formed against denatured or dissociated HC were not [6–9, 26]. Similarly, for HLA-II antigens using three different lots of LS beadsets, Grenzi et al. [10] observed anti-HLA-II reactivities for 141 sera using the LS beadsets coated with HLA-DRB1*09:01, DRB3*01:01, DRB3*02:02, DRB3*03:01, DPB1*02:01, DPB1*20:01 and DPB1*28:01. Sera reacting to LS beads failed to react with the native cell surface HLA (e.g., HLA-DRB1*09:01) in flow crossmatch and in absorption/elution experiments, suggesting that that the HLA-DRB on the beadsets may exist as denatured variants. These findings are most relevant because the reactivity of a transplant candidate’s serum against “denatured” variants may result in “inappropriate assignment of unacceptable antigens” [11]. Therefore, it is critical that clinical HLA laboratories should utilize beadsets devoid of monomeric variants of HLA or the “denatured HLA”.

Eversince SAB assay replaced cell-based assays, HLA antigen coated LABScreen (LS) beadsets were extensively used to monitor HLA antibodies in clinical transplantation. Recently, using three unique mAbs (W6/32, HC-10 & TFL-006) at the same concentration and ratios, we have [12] documented that the LS beadsets not only carry intact native HLA-I (β2M-associated, peptide-associated HC), but also peptide-free, β2M-associated HC and β2M-free HC. HC-10 positivity denotes peptide-free β2M-associated HC, which is found on both LS and LC beadsets but at a lower level on LC. TFL-006 positivity denotes the presence of β2m-free HC. Table 7 illustrates the density (MFI) measured as normalized MFI of β2-microglobulin free HLA-I molecules (monomeric variant of HLA-I or also commonly referred to as “denatured HLA”) in the LS (Lot # 10) and LC (Lot # 3005613) beadsets. Most importantly, the HLA-I polyreactive mAb TFL-006 did not react with LC beadsets [13], indicating that they are devoid of β2m-free HC variants. In all possibility, it appears that the vendors (Immucor Inc) of LC beadsets have succeeded in generating intact, native, trimeric recombinant HLA molecules, devoid of monomeric contaminants, to coat on the solid matrix microbeads. Since mAb TFL-006 has the potential to recognize “shared” or “public” epitopes common to most of the HLA-I antigens [22, 23], the mAb has become a superior diagnostic agent to quality control the HLA-I molecules without monomeric variants on the beadsets. Such a similar mAb with potential to recognize shared or public epitopes common to most of HLA-II antigens would be highly valuable for quality control of HLA-II coated beadsets.

**Table 7:** The density measured as normalized MFI of β2-microglobulin free HLA-I molecules (monomeric variant of HLA-I or also commonly referred to as “denatured HLA”) in the beadsets LS and LC

Normalized MFI after mAb								
Antigen	LS (Lot# 10)	LC (Lot # 3005613)	Antigen	LS (Lot# 10)	LC (Lot # 3005613)	Antigen	LS (Lot# 10)	LC (Lot # 3005613)
	(20 μg/mL)		B*07:02	862	0	Cw*01:02	4066	1
NC	0	0	B*07:03	N/A	0	Cw*02:02	7446	20
PC	12	0	B*08:01	1226	0	Cw*03:02	2889	N/A
A*01:01	933	0	B*13:01	6915	N/A	Cw*03:03	2458	0
A*02:01	339	0	B*13:02	2514	12	Cw*03:04	4504	0
A*02:02	N/A	0	B*14:01	7805	0	Cw*04:01	3337	8
A*02:03	1018	0	B*14:02	1831	3	Cw*04:03	N/A	0
A*02:05	N/A	0	B*15:01	335	0	Cw*05:01	9124	24
A*02:06	1230	N/A	B*15:02	1935	0	Cw*06:02	5644	4
A*03:01	193	0	B*15:03	1822	0	Cw*07:01	N/A	27
A*11:01	4782	0	B*15:10	1010	N/A	Cw*07:02	8702	97
A*11:02	537	0	B*15:11	5165	N/A	Cw*08:01	6090	1
A*23:01	133	0	B*15:12	770	0	Cw*08:02	N/A	3
A*24:02	716	0	B*15:13	3135	0	Cw*12:02	N/A	10
A*24:03	2516	0	B*15:16	3076	0	Cw*12:03	3562	N/A
A*25:01	194	0	B*15:18	N/A	0	Cw*14:02	3937	0
A*26:01	2221	0	B*18:01	3096	0	Cw*15:02	4465	4
A*29:01	1017	0	B*27:03	N/A	0	Cw*16:01	4648	0
A*29:02	778	0	B*27:05	634	0	Cw*17:01	8296	10
A*30:01	1496	0	B*27:08	1659	0	Cw*18:01	N/A	0
A*30:02	1135	N/A	B*35:01	6128	0	Cw*18:02	8015	N/A
A*31:01	396	0	B*35:08	N/A	0			
A*32:01	515	0	B*37:01	2650	0			
A*33:01	1038	0	B*38:01	2521	0			
A*33:03	554	0	B*39:01	704	0			
A*34:01	2616	N/A	B*40:01	3429	0			
A*34:02	1535	0	B*40:02	2697	0			
A*36:01	1353	0	B*40:06	10,684	N/A			
A*43:01	2479	0	B*41:01	3739	0			
A*66:01	1886	0	B*42:01	347	0			
A*66:02	1454	0	B*44:02	3650	0			
A*68:01	713	0	B*44:03	1829	0			
A*68:02	1185	0	B*45:01	1736	0			
A*69:01	3128	0	B*46:01	3572	0			
A*74:01	652	0	B*47:01	2152	0			
A*80:01	3132	0	B*48:01	3262	10			
			B*49:01	1554	0			
			B*50:01	1799	0			
			B*51:01	2461	8			
			B*51:02	2695	N/A			
			B*52:01	2146	0 5442			
			B*53:01	5442	2			
			B*54:01	1662	0			
			B*55:01	2519	0			
			B*56:01	3662	0			
			B*57:01	2089	0			
			B*57:03	2260	N/A			
			B*58:01	5268	0			
			B*59:01	3553	1			
			B*67:01	406	0			
			B*73:01	1423	2			
			B*78:01	2996	0			
			B*81:01	1525	0			
			B*82:01	3442	N/A			
			B*82:02	N/A	0			

The results of this investigation are unique as they reveal not only the number of antigens recognized (MFI > 500) by the antibodies in the sera (tested at dilution 1/10) but also the strength (MFI) of the antibodies against both classes of HLA antigens, which are often higher with LS than with LC beadsets. For HLA-I, MFIs observed on LS beadsets were higher than those for LC in 16 out of 18 sera tested with *IgHPolyFab.* For HLA-II, 14 out of 18 sera monitored with *FcMonoIgG* were higher with LS beads. For example, there are antibodies specific for a subset of antigens (for example, DRB1*04:01 and DRB*1*04:03 in MGH-001) that are clearly detected by LS only and show no reactivity with LC. See also sera groups 1A, 1B for HLA-I and HLA-II for further examples. The LC beads were either totally non-reactive or showed very low reactivity to several HLA antigens in contrast to LS beadsets, with both secondary antibodies These results together with higher number HLA-I and II reactive IgGs detected on the LS than on the LC beadsets, as well as the earlier comparative study carried out with LS and iBeads [8, 9, 12, 13, 26], suggest the higher prevalence of antibodies reacting to “conformational” HLA variants are bound on to the LS beadsets.

Furthermore, the intraindividual variability in both the breadth of positivity and the intensity of individual reactions between LS and LC is compounded by the secondary antibodies used to detect the primary antibody bound to the beadsets. Throughout the literature, polyclonal F(ab)2 fragments raised against either the IgH constant region (provided with LS kits) or Fc-Gamma constant HC (provided with LC kits) are used. The fluorescent phycoerythrin molecule conjugated to the secondary antibody is detected by Luminex and is reported as the mean fluorescence intensity (MFI). The MFIs obtained with the secondary antibody will be directly proportional to the primary antibody only if the secondary antibody binds to the primary antibody at a one to one ratio [19]. If multiple polyclonally derived F(ab)_2_ bind to one or more constant region domains on the HC of the primary antibody, the signal will be amplified depending on the number of polyclonal Fab binding to different epitopes on IgH, because polyclonal F(ab)s may bind to different epitopes on IgH. In this regard, *FcMonoIgG* binds to Fc-gamma HC in one to one ratio. Indeed, comparing *IgHPolyFab* versus *FcMonoIgG* on LS beadsets in an earlier study [19], as well as in this investigation, we observe that a greater number and MFIs of class I HLA antigens with *IgHPolyFab* than with *FcMonoIgG,* evidently due to multiple F(ab)_2_ binding to one primary antibody. The MFI of several (though not all) anti-HLA-I antibodies detected using *IgHPolyFab* were more often higher than that recognized by *FcMonoIgG* for the following sera: MGH-001/-006/-007/-011/-014/-020 and -024. Overall, in all the sera tested, the total number of HLA-A (LS 43, LC38, *p* < 0.0005) and HLA-B (LS 53, LC 26, *p* < 0.06) antigens with *IgHPolyFab* was significantly higher than that recognized by *FcMonoIgG.*

However, for anti-HLA-II antibodies, only MGH-024 showed higher MFI with *IgHPolyFab,* whereas all other sera revealed higher MFI with *FcMonoIgG.* This finding to some extent is in contrast to anti-HLA-I antibodies. *FcMonoIgG,* in contrast to *IgHPolyFab,* recognized a significantly higher number of antibodies reacting to HLA-DR and DQ antigens on LS beadsets. Similarly, *FcMonoIgG,* compared to *IgHPolyFab* recognized high numbers of antibodies reacting to DQ antigens on LC. The number of antigen-reactive antibodies at the DRB locus is significantly higher on LS with *FcMonoIgG (n* = 61) than with *IgHPolyFab* (*n* = 42) and so also the number of antibodies reacting at the DQ locus (39 vs 32 alleles). We have observed this phenomenon earlier [19] and suggested that the *IgHPolyFab* may not be capable of binding to the heavy chain of IgH of primary antibody that is bound to the HLA on beads. It was attributed to the aggregation of serum antibodies on the beads due to increased density of serum antibodies added on to the beads, in general [27–30] and anti-HLA antibodies in particular [29], consequent to a high level of antigen sensitization. Even if the beads are washed well, aggregation of IgG with or without adherence of IgM or immune complexes to the aggregates cannot completely be removed. This is one of the reasons to titrate the antibody before applying to the solid matrix. Recently [19], we have examined how the *IgHPolyFab* differs from *FcMonoIgG* in elucidating the prozone effect or low vs high titer using LABScreen beadsets. Figure 4A-C in this earlier report [19] illustrated the distinct disparity in the serum titrimetric profiles of MFIs and the prozone effects for anti-HLA-I and HLA-II antibodies tested using LS beadsets with *IgHPolyFab* and *FcMonoIgG*. The results of the present investigation clarify that *studying the prozone effect or low or high titers using LS beadsets is not of much clinical relevance since the HLA antibodies recognized using LS beadsets consists of a mixture of antibodies reacting to both intact HLA and denatured monomeric variants of HLA* and hence the titrimetric analysis of sera were not carried out in this study. Furthermore, the cohort of sera were not suitable for detailed cost-prohibitive titrimetric investigation for the cohort is not an homogenous entity of ESRD, as described in Material and Methods section.

Other investigators have observed a similar increase in the density of serum IgG concentration against alloantigens, autoantigens and anti-idiotype antigens even before transplantation [27–30]. We hypothesized that when antibodies are at low density (as evidenced by low MFI (< 3000) of anti-HLA antibodies), the IgH is exposed for multiple binding of *IgHPolyFab* to amplify the fluorescent intensity. It was noted that the MFIs obtained with sera or IgG purified from the sera of normal individuals or with IVIg (free of IgM or complement proteins or immune complexes) detected with *IgHPolyFab* is higher than that of *FcMonoIgG* due to the lower density of serum IgG antibodies [19]. At a higher density of antibodies, the titrimetric profile showed higher MFI (> 3000) or greater Fc-affinity with *FcMonoIgG* [19]. In addition to the density of antibodies, there could be other independent factors that may impede binding of *IgHPolyFab.* Steric interference of IgM in anti-HLA IgG detection (with *IgHPolyFab*) has also been observed with flow beads assays [19].

Another potential contributing factor is complement interference, which can result in falsely low MFI values (the “prozone” phenomenon) [14–18] and fluctuations in MFI [15] when testing for anti-HLA antibodies with *IgHPolyFab* in the SAB assay. We did not investigate the influence of complement in this sera-cohort as it is not a homogenous entity of ESRD patients, as described earlier. Using reasonably well-defined sera-cohort from another center, we have examined the binding of C1q and C3d to the anti-HLA antibodies bound to the two beadsets (LS & LC) from different vendors and monitored with two different secondary antibodies (manuscript in preparation). The results reveal when the antibodies were detected only on HLA molecules coated on LS but not on to the corresponding molecules on LC, obviously due to antibodies reacting to the monomeric variants on LS, C1q binding was observed only on LS but not on LC. When antibodies were detected abundantly or only on HLA molecules coated on LC but not on to the corresponding molecules on LS, indicative of prevalence of antibodies against intact native trimeric HLA, C1q (as well as C3d) binding was observed only on LC but not on LS beadsets. These observations confirm the complement influence on both antibodies binding to intact native trimeric HLA as well as to monomeric or denatured variants of HLA. The prozone phenomenon due to complement interference does exist, particularly when sera are tested at neat or low dilutions [14], as is done in most of the investigations. Observations carried out on post-transplant sera of patients using “denatured HLA-admixed” LS beadsets and *IgHPolyFab*, as detection antibody, lead many US investigators to contend that antibodies against HLA class I molecules may not be clinically relevant to the extent of anti-HLA-II antibodies, in organ transplantation. This report, as well as the manuscript in preparation using another sera-cohort, emphasizes the need to examine the sera using LC beadsets with *FcMonoIgG* as detection antibodies. Indeed, recently Kamburova et al. [31] including Claas, Otten and Spierings from Netherlands have investigated the impact of pretransplant DSA, assessed using LC beadsets but with *IgHPolyFab*, on long-term graft survival in 3237 deceased- and 1487 living-donor kidney transplantations to observe the clinical relevance of anti-HLA-I antibodies are indeed comparable to that of anti-HLA-II antibodies.

The intraindividual variability also changes the percentage of cPRA, which is critical for assignment of deceased donor organs. Examining cPRA for the two beadsets tested with two secondary antibodies in sera groups 1A, 1B, 2A, and 2B, we found that the higher percentage of cPRA is parallel with the number of antibodies on LS beadsets in contrast to LC. In these sera groups, MFI rarely exceeded 2500. Similarly higher numbers of anti-HLA IgG paralleled the high percentage of cPRA, when tested with *IgHPolyFab* for class I, and with *FcMonoIgG* for class II. Such parallel association of the number of HLA and percentage cPRAs may lead, as predicted by Michel et al. [11], to “inappropriate assignment of unacceptable antigens during transplant listing” and deny living or deceased donor organs to the patients in the waiting list.

In this study, there were a number of sera with anti-HLA reactivity detected only with LS beads, including one patient with a cPRA of 0% when tested by LIFECODES and 99.5% with LABScreen. Detection of false positive anti-HLA antibodies could result in inappropriate designation of unacceptable antigens, and potentially deny a patient access to compatible organs. The ultimate result could be the inappropriate administration of costly and potentially toxic desensitization procedures with detrimental consequences. The potential infectious side-effects of such desensitization have been well described [30, 32–38].

In summary, this study confirms significant inter- and intraindividual variability in the number and MFI of HLA antibodies monitored using single antigen beads from two vendors and two secondary antibodies. The most commonly used methodology, LS beadsets with *IgHPolyFab*, resulted in a significantly greater number of IgG antibodies against HLA-I antigens being deemed unacceptable, as well as with significantly higher MFIs, compared to LC beadsets. In the case of HLA-II antigens, LS beadsets again resulted in higher number of unacceptable antigens compared to LC beadsets, but in this instance, *FcMonoIgG* as secondary antibody resulted in higher MFIs than *IgHPolyFab*. Furthermore, higher reactivity of LS beadsets, with either of the second antibodies is not surprising since LS beadsets contain “denatured” or “conformational” variants of HLA-I antigens compared to LC beadsets [12, 13], which contained only β2m-associated HC of HLA. The findings of Grenzi et al. [10] suggest that this could be true for HLA-II antigens too. The negative impact of the higher reactivity and false positivity based on the brand of beadsets and the nature of the secondary antibody is critically important, as it may result in the denial of otherwise acceptable organs and inappropriate desensitization procedures.

Our study has obvious limitations. Mainly, we have examined a very small number of patients from a single center. Furthermore, the cohort of sera may consist of non-clinically relevant allo-HLA reactivity and the cohort is not a homogenous entity of ESRD patients, as described in the “Material and Methods” section. There was no gold-standard to verify the accuracy of the respective assays in terms of predicting true pathogenicity following transplantation. More observations are clearly needed to compare these differing methodologies with their ability to accurately predict crossmatches and, most importantly, long-term allograft outcomes.

## Abbreviations

cPRA: Calculated panel reactive antibody
β2M: β2-Microglobulin
FcMonoIgG: Fc-specific monoclonal IgG secondary antibody
HC: Heavy chain
HLA: Human leukocyte antigen
IgHPolyFab: IgG heavy chain binding polyclonal antibody fragment of secondary antibody
LS: LABScreen
LC: LIFECODES
MFI: Mean fluorescent intensity
SAB: Single antigen bead assays

## Footnotes

1 Ravindranath et al. [13] has inadvertently indicated that Lot # 03203F (expiration date 3/31/2014) was used, but on later verification corrected the Lot number as 12235B (expiration date 02/2017) and the assay was performed on 05/23/2016.
